# Estratégias Econômicas e Sociais para Anticoagulação de Pacientes com Fibrilação Atrial

**DOI:** 10.36660/abc.20200921

**Published:** 2022-01-01

**Authors:** Andressa Zulmira Avila Guerrero, Enia Lucia Coutinho, Marcos Bosi Ferraz, Claudio Cirenza, Marcelo Cincotto Esteves dos Santos, José Roberto Ferraro, Angelo Amato Vincenzo de Paola

**Affiliations:** 1 Universidade Federal de São Paulo São Paulo SP Brasil Universidade Federal de São Paulo, São Paulo, SP- Brasil

**Keywords:** Custos de Cuidados de Saúde, Anticoagulantes, Varfarina, Fibrilação Atrial

## Abstract

**Fundamento:**

A fibrilação atrial é um problema de saúde pública associado com um risco cinco vezes maior de acidente vascular cerebral e mortalidade. A análise de custos é importante para a introdução de novas terapias, e deve ser reconsiderada em situações especiais, tais como a pandemia do coronavírus em 2020.

**Objetivo:**

Avaliar os custos (em um período de um ano) relacionados à terapia anticoagulante e a qualidade de vida de pacientes com fibrilação atrial tratados em um hospital público universitário.

**Métodos:**

Os custos do paciente foram aqueles relacionados à anticoagulação e calculados pela média de custos mensais da varfarina ou de anticoagulantes orais diretos (DOACs). As despesas não médicas, como alimentação e transporte, foram calculadas a partir de dados obtidos de questionários. O questionário brasileiro SF-6D foi usado para medir a qualidade de vida. Valores p<0,05 foram considerados estatisticamente significativos.

**Resultados:**

A população do estudo consistiu em 90 pacientes, 45 em cada braço (varfarina vs. DOACs). Os custos foram 20% mais altos no grupo dos DOACs (US$55 532,62 vs. US$46 385,88), e principalmente relacionados ao preço dos medicamentos (US$23 497,16 vs. US$1903,27). Os custos hospitalares foram mais altos no grupo da varfarina (US$31 088,41 vs $24 604,74), e relacionados às visitas ao ambulatório. Ainda, as despesas não médicas foram duas vezes maiores no grupo varfarina ($13 394,20 vs $7 430,72). A equivalência de preço entre os dois medicamentos seria alcançada por uma redução de 39% no preço dos DOACs. Não foram observadas diferenças quanto à qualidade de vida.

**Conclusões:**

Os custos totais foram mais altos no grupo de pacientes tratados com DOACs que no grupo da varfarina. No entanto, uma redução de cerca de 40% no preço dos DOACs tornaria viável a incorporação desses medicamentos no sistema de saúde público brasileiro.

## Introdução

A fibrilação atrial (FA) não valvar é um problema de saúde pública associado a um risco cinco vezes maior de acidente vascular cerebral (AVC) e mortalidade.^1–4^ A varfarina é amplamente utilizada nessa condição,^5–7^ e os pacientes geralmente necessitam de consultas ambulatoriais frequentes para se alcançar um tempo na faixa terapêutica (TTR, *time in therapeutic range*) adequado.^8–11^ O controle laboratorial e as visitas ao ambulatório são não apenas uma carga econômica, como também uma questão de distanciamento social, ambos fatores importantes desde que a Organização Mundial da Saúde declarou a doença do coronavírus 2019 (COVID-19) como uma emergência de saúde pública internacional. Estudos sobre medidas preventivas e o controle efetivo de infecções durante a pandemia foram realizados.^12,13^

Os anticoagulantes orais diretos (DOACs), são um avanço importante na terapia de anticoagulação na FA, facilitaram o controle clínico, com boa eficácia e segurança.^14–16^ No cenário atual de pandemia, a terapia anticoagulante com DOACs é adequada uma vez que prescinde de visitas frequentes a serviços de saúde, favorecendo o distanciamento social.^17^ Contudo, os DOACs ainda não estão disponíveis no sistema de saúde no Brasil. Somente 30% dos pacientes no país possuem seguro saúde privado e acesso aos DOACs para prevenção de AVC.^18^

Recursos limitados dificultam a implementação de novas tecnologias, o que representa um problema na saúde pública. Avaliações da economia ajudam a aliviar a carga de recursos escassos, melhorando a eficácia alocativa do financiamento dos serviços de saúde.^19,20^

Este estudo teve como objetivo avaliar os custos relacionados à terapia anticoagulante (varfarina vs. DOACs) no manejo da FA, utilizando custos do mundo real em pacientes tratados em um hospital universitário pelo sistema público de saúde.

## Métodos

### População do estudo

Este foi um estudo observacional e retrospectivo. Calculou-se um tamanho amostral de 89 pacientes com base no ambulatório de FA da Universidade Federal de São Paulo (680 pacientes), em que a prevalência de varfarina foi de 92% e a de DOACs de 8% somente. Um total de 90 pacientes foram recrutados. A amostragem foi realizada por conveniência para equilíbrio dos braços de tratamento (por exemplo, para cada paciente em uso de DOAC incluído, um paciente em uso de varfarina foi convidado a participar). O estudo foi aprovado pelo Comitê de Ética da Universidade Federal de São Paulo (#79785717.2.0000.5505). Os pacientes que não compreenderam ou não aceitaram a assinar o termo de consentimento não foram incluídos.

Todos os pacientes foram incluídos em 2018 e os custos foram analisados no período de acompanhamento de 12 meses. A coleta de dados a partir dos prontuários médicos não afetou a frequência de visitas dos pacientes ao hospital ou a dosagem de varfarina ou DOACs, estabelecidas a critério da equipe de anticoagulação. Para a coleta de dados, desenvolvemos dois questionários utilizando o REDCAP (Research Electronic Data Capture),^21^ que consiste em uma aplicação *web* segura para a criação e gerenciamento *online* de pesquisas e banco de dados:

1. **Questionário clínico/econômico:** incluiu itens a respeito de dados demográficos (idade, sexo, profissão, educação), história clínica (comorbidades), e dados econômicos (salário, renda familiar, tipo de transporte, tempo de viagem).
2. **Operacional:** quantificou os custos individuais dos participantes na instituição. A soma dos valores foi feita por meio do Key Performance Indicators for Health (KPIH), um sistema de cálculo e análise de custos de nossa instituição.

### Custos

O estudo comparou os custos de duas intervenções similares para identificar a de menor custo enquanto se mantinha um nível desejável de qualidade. Os custos foram classificados em duas categorias – custos dos pacientes e custos do hospital.^22^

#### Custos do paciente

Os custos médicos do paciente incluíram os custos relacionados à anticoagulação e foram calculados pelos custos mensais médios com varfarina ou DOACs pagos e identificados pelo paciente. Os custos não médicos dos pacientes foram calculados a partir de dados fornecidos no questionário, tais como os custos com refeições/bebidas, transporte, e outros gastos. Para o cálculo com transporte, foi considerado o meio de transporte utilizado por cada paciente no dia da visita ao hospital. Para os pacientes que necessitaram de transporte público, consideramos o preço da passagem/ticket de ônibus e o número de passagens/tickets usados por dia. Para os pacientes que utilizaram carro próprio, considerou-se a distância entre a casa e a clínica de anticoagulação e o consumo de combustível.

Os custos foram calculados usando os preços de janeiro de 2020 e corrigidos de acordo com o índice de preços no consumidor, e convertidos a dólares americanos em 09 de abril de 2020 (1 dólar americano = 5,10 reais). Neste estudo, todos os custos foram expressos em dólares americanos (US$).

O custo de oportunidade, que se referiu à quantidade de renda que o paciente e/ou familiar deixou de ganhar no trabalho durante o estudo, foi incluído na análise de perda de produtividade. A perda de produtividade no trabalho foi calculada com base nos dias de absenteísmo e perda no salário.

#### Custos hospitalares

Os custos hospitalares referiram-se aos serviços oferecidos a cada paciente, e coletados por meio do sistema KPIH. Esse consiste em um indicador de desempenho e quantifica os custos diretos e indiretos de cada departamento. Os custos médicos diretos incluíram os relacionados com medicamentos, internações, sala de emergência, visitas ao ambulatório, exames laboratoriais e de imagem. Os custos indiretos incluíram os gastos gerais e administrativos (por exemplo, despesas com departamento de contabilidade e departamento pessoal), e despesas incorridas em áreas do hospital não geradoras de renda que devem ser alocadas sobre as áreas geradoras de renda do hospital.

### Qualidade de vida (SF-6D)

Questionário de qualidade de vida de 36 itens (SF-36) é um questionário genérico de qualidade de vida composto de 36 itens/questões, distribuídos entre oito domínios, resumidos em um componente físico e um componente mental. Em 2002, Brazier et al.^23^ revisaram o SF-36 e desenvolveram um novo índex que estabeleceu uma nova classificação de status de saúde usando seis domínios (SF-6D). Uma versão brasileira do SF-6D possibilita gerar valores de utilidade, e escores mais altos indicam uma melhor qualidade de vida. O SF-6D descreve saúde em seis dimensões (função física, limitação nas funções, função social, dor, saúde mental, e vitalidade), de dois a seis níveis de gravidade, descrevendo, assim 18 mil estados de saúde possíveis.^24^

#### Análise estatística

Para análise estatística dos dados, as variáveis quantitativas foram expressas em média e desvio padrão (DP) e as variáveis qualitativas como valores absolutos e porcentagens. A normalidade da distribuição dos dados quantitativos foi avaliada pelo teste de Shapiro-Wilk. Para comparações entre os grupos, o teste t de Student não pareado foi usado para dados quantitativos, e o teste do qui-quadrado para dados qualitativos. Valores p<0,05 foram considerados estatisticamente significativos. Todas as análises foram realizadas pelo programa SPSS Statistics, versão 25.0 (IBM Corp., Armonk, NY, EUA).

## Resultados

### A) Características sociodemográficas

A população do estudo foi composta por 90 pacientes, 45 em cada braço do estudo (varfarina vs. DOACs; Tabela 1). Todos os participantes no braço de antagonistas da vitamina K (AVK) usavam varfarina. Os DOACs utilizados pelos pacientes foram distribuídos da seguinte maneira: rivaroxabana 70%, apixabana 18%, dabigatrana 10%, e edoxabana 2%. Os fatores de risco e condições basais estão descritos na Tabela 1. As médias do escore CHA2DS2-VASc foi três, e a média do escore HAS-BLED foi um.

**Tabela 1:** Características sociodemográficas e fatores de risco nas condições basais dos pacientes com fibrilação atrial em uso de varfarina e anticoagulantes orais diretos (DOACs) atendidos no ambulatório da Universidade Federal de São Paulo em 2018

	varfarina (n = 45)	DOACs (n = 45)	Valor p
Idade (anos)	68 ± 9	72 ± 10,5	0,07
Mulheres (%)	51%	53%	0,08
Ensino fundamental, n (%)	32 (71)	24 (53)	0,08
Ensino superior, n (%)	12 (27)	21 (47)	0,04
Aposentados, n (%)	34 (76)	36 (80)	0,06
Salário (US$)	304,03 ± $211,34	379,83 ± $300,05	0,12
Necessidade de transporte público, n (%)	37 (84)	23 (52)	0,01
Tempo de viagem por consulta (hr.: min.)	2:50 ± 1:24	2:55 ± 3:06	0,45
Hipertensão, n (%)	36 (82)	27 (60)	0,03
Diabetes mellitus, n (%)	14 (31)	11 (24)	0,48
Insuficiência cardíaca, n (%)	9 (20)	8 (17)	0,86
Infarto do miocárdio, n (%)	6 (13)	6 (13)	0,10
AVC/Ataque isquêmico transitório, n (%)	6 (13)	5 (11)	0,74

*As variáveis quantitativas são expressas em média ± desvio padrão, e as variáveis qualitativas em valores absolutos e porcentagens. Ensino fundamental completo é definido como a conclusão de cinco anos dessa fase escolar. Valores p < 0,05 foram considerados estatisticamente significativo. AVC: acidente vascular cerebral*

### B) Custos hospitalares, custos pessoais, e custo total

Os custos hospitalares foram maiores no grupo varfarina (Tabela 2); seis (12%) pacientes do grupo varfarina e 26 (52%) do grupo DOACs iam rotineiramente às consultas no ambulatório com um familiar acompanhante. Os custos relacionados às perdas de produtividade dos pacientes e acompanhantes estão apresentados na Tabela 3 e os custos totais na Tabela 4.

**Tabela 2:** Custos da instituição por serviços prestados ao paciente

	No.	Custos com varfarina (US$)	No.	Custos com DOACs (US$)
Exames laboratoriais	1021	2347,17	671	1467,61
Exames de imagem	208	3110,75	318	5976,94
Visitas ao hospital	688	12 778,02	348	4628,20
Sala de emergência	71	9496,54	85	7475,8
Internações	8	3355,93	10	5056,81
Total (US$)		31 088,41		24 604,74

*DOACs: anticoagulantes orais diretos.*

**Tabela 3:** Custos totais do paciente para recebimento do tratamento

		Custos com varfarina (US$) (n=45)	Custos com DOACs (US$) (n=45)
	Transporte	2790,32	1011,10
Paciente/Cuidador	Alimentação	2669,67	605,25
	Medicamentos	1903,27	23 497,16
Perda de produtividade	Paciente	7393,77	5 185,43
	Cuidador	540,43	628,94
Total		15 297,47	30 927,88

*DOACs: anticoagulantes orais diretos*.

**Tabela 4:** Despesas totais (referente a 12 meses) dos pacientes com fibrilação atrial atendidos no ambulatório da Universidade Federal de São Paulo em 2018 em tratamento com varfarina (n=45) ou anticoagulantes orais diretos (n=45)

	Custos com varfarina (US$) (n=45)	Custos com DOACs (US$) (n=45)
Custo pessoal	15 297,47	30 927,88
Custo hospitalar	31 088,41	24 604,74
Total	46 385,88	55 532,62

*DOACs: anticoagulantes orais diretos.*

### C) Visão geral dos custos baseada no Sistema Único de Saúde (SUS)

Os DOACs foram 10 vezes mais caros que a varfarina; no entanto, os custos totais do braço dos DOACs foram aproximadamente 1,2 vezes (20%) maiores. A enorme diferença entre esses dois tipos de anticoagulantes (DOACs US$23 497,16 vs. varfarina US$1903,27) pode ser muito reduzida se considerarmos os demais custos envolvidos no estudo. Nesse cenário, os custos indiretos do paciente foram relacionados à perda de produtividade, e os custos diretos a transporte e alimentação, o que contribuíram ao aumento dos custos no braço da varfarina. A diferença de US$9146,74 (Fórmula 1) poderia ser eliminada com uma redução de aproximadamente 40% (Fórmula 2) no preço dos DOACs.

**Fórmula 1 – diferença real: diferença entre os custos totais com DOACs e varfarina:**


$$\begin{array}{l} \text{DOACs total}-\text{varfarin total}\\ \text{US}\$55\;532,62-\text{US}\$46\;385,88\\ \text{Diferença real US}\$9146,74 \end{array}$$

**Fórmula 2 – Redução necessária no preço dos DOACs para torná-la comparável ao preço da varfarina:**


$$\begin{array}{l} \text{Preço dos ADOCs}-\text{Diferença real difference }\left(\text{fórmula 1}\right)=\\ \text{US}\$23\;497,16-\$9\;146,74\;=\;\$14\;350,42 \end{array}$$

(61% do preço dos AODCs ou redução de 39% no preço do medicamento)

### D) Questionário *Short Form* (SF-6D)

O SF-6D foi usado para medir a qualidade de vida dos participantes (Tabela 5). Os escores de utilidade mais baixos foram relatados pelo domínio da função social, mostrando um importante déficit social em ambos os braços. A média do escore de utilidade do SF-6D foi 0,649 para o grupo AVK vs 0,641 para o grupo DOACs (Tabela 5). Não foi observada diferença em nenhum outro parâmetro de qualidade de vida entre os grupos.

**Tabela 5:** Média de escores dos domínios do questionário de qualidade de vida SF-6D dos pacientes com fibrilação atrial atendidos no ambulatório da Universidade Federal de São Paulo em 2018 em tratamento com varfarina (n=45) ou anticoagulantes orais diretos (DOACs) (n=45)

	varfarin	DP	DOACs	DP
Função física	0,692	0,338	0,633	0,429
Limitações nas funções	0,564	0,356	0,570	0,364
Função social	0,240	0,302	0,220	0,274
Dor	0,877	0,324	0,906	0,393
Saúde mental	0,680	0,424	0,802	0,322
Vitalidade	0,446	0,236	0,448	0,220

*Os escores do SF-6D varia de 0 a 1, em uma escala onde 0 é igual ao pior estado de saúde e 1 significa o melhor estado de saúde. DOACs.: anticoagulantes orais diretos; DP: desvio padrão.*

## Discussão

A ampla utilização da varfarina no Brasil é justificada por seu baixo custo e boa eficácia. No entanto, a necessidade de controle laboratorial e variações no TTR são causas frequentes de complicações clínicas e interrupção no tratamento medicamentoso,^25,26^ o que pode tornar o manejo do paciente ainda mais desafiador.^27^ A subutilização de anticoagulantes é mais pronunciada em países de renda média, como em países da América do Sul, em que o uso chega a ser inferior a 40%.^28^

Os DOACs foram introduzidos no Brasil em 2012,^29^ com uma clara vantagem sobre outras terapias.^30–32^ Infelizmente, devido aos altos custos, os DOACs têm sido praticamente utilizados exclusivamente na prática privada. Contudo, a necessidade de testes laboratoriais e frequentes visitas ao hospital para o manejo da terapia com varfarina geram problemas logísticos que se tornaram mais críticos na pandemia da COVID-19 devido à necessidade de distanciamento social/isolamento.^2,17,33^ Tais problemas podem ser minimizados com a incorporação dos DOACs ao SUS. Uma análise de custo efetividade dá evidência de que, apesar dos custos mais elevados, os DOACs são mais vantajosos, com melhor custo efetividade que a varfarina.^28,34–39^

A avaliação econômica com foco somente na assistência financeira direta não leva em consideração alguns fatores importantes para a análise global, como transporte, alimentação, absenteísmo no trabalho, e estresse.^40,41^ O grupo da varfarina apresentou mais custos diretos (por exemplo, alimentação: US$2669,67 vs. US$605,25 para varfarina e DOACs, respectivamente; transporte: US$2790,32 vs. US$1011,10 para varfarina e DOACs, respectivamente), bem como um maior número de visitas e testes laboratoriais (varfarina US$1709 vs. DOACs US$1019). No entanto, nosso estudo mostrou que os custos totais do tratamento com DOACs (US$55 532,62) foram mais altos que com varfarina (US$46 385,88). Essa diferença no preço total foi diretamente relacionada ao preço dos DOACs (DOACs US$23497,16 vs. varfarina US$1903,27).

Os DOACs não são atualmente cobertos pelo SUS principalmente por seu elevado preço. Estudos locais com foco não somente em custos institucionais como também em custos sociais são necessários. O manejo da varfarina é difícil; muitos clínicos gerais são céticos em prescrever ou manter prescrições prévias de AVK. Nosso estudo reforça a ideia de que os DOACs podem ser fornecidos pelo serviço público de saúde. Silva et al.,^42^ em um estudo sobre a qualidade do controle da anticoagulação em pacientes com FA não valvar, tratados com varfarina em um serviço de saúde privado, relataram que mais de 60% dos pacientes estavam abaixo do alvo desejado (em termos de TTR), o que se associou com maiores custos.

Com base nas nossas análises, o preço “ideal” dos DOACs seria US$14 350,42 (fórmula 2). Nesta situação, o controle de anticoagulação com varfarina e DOACs teria custos comparáveis. Pacientes que não requerem monitoramento frequente estão mais propensos a praticar o distanciamento social, requerido pelas autoridades sanitárias durante a pandemia da COVID-19.^40^ Nesse contexto, seria muito desejável o desenvolvimento de uma abordagem viável de incorporação de DOACs nas políticas públicas de saúde, contribuindo para um sistema de saúde único e justo. Reduções nos custos no grupo DOACs são a única possibilidade de redução de custo nesse cenário. Uma estratégia simples para isso seria a redução do preço dos DOACs, calculado para esse propósito neste estudo.

O reembolso dos gastos no SUS é inadequado, com pouca transferência de pagamentos para a saúde pública. Assim, medidas administrativas são necessárias para promover um equilíbrio racional dos custos e acuidados acessíveis e conscientes. Sugerimos que algumas intervenções nos preços poderiam gerar um retorno econômico positivo, e a prestação eficiente de serviços de saúde, levando a um cenário viável para o uso de DOACs na prática clínica.

Interessante notar que um grande número de consultas não influenciou a qualidade de vida relacionada à saúde no grupo varfarina. Provavelmente, o alto grau de humanismo de nossa equipe de anticoagulação na FA tenha compensado a falta de apoio social e desconforto causados pelas visitas repetitivas ao hospital, conforme descrito em estudos realizados em outros países.^43–45^ O fornecimento de DOACs a pacientes com FA é justificado e necessário não apenasdurante uma pandemia, como também em cenários clínicos e epidemiológicos normais.

### Limitações

Nosso estudo apresentou algumas limitações. O estudo não foi randomizado, e desfechos como AVC, mortalidade e adesão não foram analisados. As despesas foram analisadas durante um período de 12 meses, e possivelmente um acompanhamento de longo prazo possa revelar mais benefícios.

## Conclusão

Os custos no grupo DOACs mostraram-se maiores que no grupo varfarina. Custos não medicamentosos são importantes cargas no grupo varfarina, demandando estratégias econômicas em saúde racionais para o manejo da doença. Uma redução de 40% no preço das DOACs pode ser importante para a incorporação desses medicamentos no SUS, uma política viável durante a pandemia da COVID-19.
