# Supplementary material for: Estratégias Econômicas e Sociais para Anticoagulação de Pacientes com Fibrilação Atrial
Source: Arq Bras Cardiol. 2022 Jan 1;118(1):88–94. [Article in Portuguese] doi: 10.36660/abc.20200921 (PMC8959044; doi:10.36660/abc.20200921)
Supplement: Translation

# Translation: Economy- and Social-Based Strategies for Anticoagulation of Patients with Atrial Fibrillation

**Authors:** Andressa Zulmira Avila Guerrero, Enia Lucia Coutinho, Marcos Bosi Ferraz, Claudio Cirenza, Marcelo Cincotto Esteves dos Santos, José Roberto Ferraro, Angelo Amato Vincenzo de Paola

**Affiliations:** 1 São Paulo SP Brazil Universidade Federal de São Paulo, São Paulo, SP- Brazil

**Keywords:** Health Care Costs, Anticoagulants, Warfarin, Atrial Fibrillation

## Abstract

**Background:**

Atrial fibrillation is a public health problem associated with a fivefold increased risk of stroke or death. Analyzing costs is important when introducing new therapies and must be reconsidered in special situations, such as the novel coronavirus pandemic of 2020.

**Objective:**

This study aimed to evaluate the costs related to anticoagulant therapy in a one-year period, and the quality of life of atrial fibrillation patients treated in a public university hospital.

**Methods:**

Patient costs were those related to the anticoagulation and calculated by the average monthly costs of warfarin or direct oral anticoagulants (DOACs). Patient non-medical costs (eg., food and transportation) were calculated from data obtained by questionnaires. The Brazilian SF-6D was used to measure the quality of life. P-values < 0.05 were considered statistically significant.

**Results:**

The study population consisted of 90 patients, 45 in each arm (warfarin vs direct oral anticoagulants). Costs were 20% higher in the DOAC group ($55,532.62 vs $46,385.88), and mainly related to drug price ($23,497.16 vs $1,903.27). Hospital costs were higher in the warfarin group ($31,088.41 vs $24,604.74) and related to outpatient visits. Additionally, non-medical costs were almost twice higher in the warfarin group ($13,394.20 vs $7,430.72). Equivalence of price between the two drugs could be achieved by a 39% reduction in the price of DOACs. There were no significant group differences regarding quality of life.

**Conclusions:**

Total costs were higher in the group of patients taking DOACs than those taking warfarin. However, a nearly 40% reduction in the price of DOACs could make it feasible to incorporate these drugs into the Brazilian public health system.

## Introduction

Nonvalvular atrial fibrillation (AF) is a public health problem associated with a fivefold increased risk of stroke and mortality.^1–4^ Warfarin is largely used in this condition, ^5^ and patients usually need frequent outpatient visits to achieve the optimal time in therapeutic range (TTR). Laboratory control and outpatient visits are not only an economic burden but also a social distancing-related issue, both important factors after the World Health Organization declared the coronavirus disease-19 (COVID-19) as an international public health emergency. Studies on preventive measures and the effective control of infections during the COVID-19 pandemic have been carried out.^12,13^

Direct oral anticoagulants (DOACs), a major advancement in anticoagulant therapy for AF, have made clinical control easier, with good efficacy and safety.^14–16^ In the current pandemic scenario, anticoagulation therapy with DOACs is adequate as it dispenses with frequent visits to health services, favoring social distancing.^17^ However, DOACs are not available in the public health system in Brazil yet. It has been reported that only 30% of patients in Brazil have private health insurance and access to DOACs for stroke prevention.^18^

Limited resources hamper the implementation of new technologies and represent a real problem in public health. Economic evaluations help to alleviate the burden of scarce resources by improving the allocative efficiency of health care financing.^19,20^

This study aimed to evaluate the costs related to anticoagulant therapy (warfarin vs DOACs) in the management of AF, using real-world costs in patients treated in a university hospital by the public health system.

## Methods

### Study Population

This was an observational and retrospective study. An ideal sample size of 89 patients was calculated based on the AF outpatient clinic at the Federal University of Sao Paulo (680 patients), where the prevalence of warfarin was 92% while that of DOACs was only 8%. A total sample of 90 patients was recruited. Sampling was made by convenience for balance of treatment arms and sample homogenization (e.g for each patient taking DOACs included, a patient taking warfarin was invited to participate). The study was approved by the Institutional Review Board of Federal University of Sao Paulo (#79785717.2.0000.5505). Patients who failed to understand and/or refused to sign the informed consent form were not recruited.

All patients were included in 2018 and costs were analyzed during their last 12 month-period. The data-gathering for this study from health records did not affect the frequency of hospital visits, warfarin or DOACs dosage, which were at the anticoagulation team's discretion. For data collection, we developed two questionnaires using Research Electronic Data Capture (REDCAP),21 which is a secure web-based application for creating and managing online surveys and databases, as follows:

1. **Clinical/Economical:** This included items concerning demographic data (age, gender, occupation, education), medical history (comorbidities), and patient economic data (salary, household incoming, type of transport used, time spent traveling).
2. **Operational:** Quantified the individual costs of participants in the institution. The sum of the values was made using the Key Performance Indicators for Health (KPIH), a system for calculating and analyzing costs in our institution.

### Costs

This study compared the costs of two similar interventions to ascertain which is less expensive while maintaining a desired level of quality. The costs were classified into 2 categories: patient and hospital costs.^22^

#### Patient costs

Patient medical costs included costs related anticoagulation and were calculated by the average monthly cost of warfarin or DOACs paid and identified by the patient. Patient non-medical costs were calculated from data provided in the patient questionnaire, such as the costs of meals/drinks, transportation, and other expenses. To calculate transportation costs, the means of transport used by each patient on the day of hospital visit was considered. For those who needed public transportation, we used the price of the bus ticket and the number of tickets used per day. For patients who used their own car, we used the distance between their home and the anticoagulation clinic and calculated the fuel consumption.

The costs were calculated using January 2020 prices and corrected in accordance with the consumer price index and converted to US dollars (USD) on April 9, 2020 (USD 1 = BRL 5.10). In this study, all costs are expressed in US dollars.

Opportunity cost, referred as the amount of income that the patient and/or family caregivers missed out during the study, was included in productivity loss analysis. Loss of productivity at work was calculated based on the days of absenteeism and lost wages.

#### Hospital costs

Hospital costs referred to the services provided for each patient and were collected using the KPIH system. This is a performance indicator and quantifies the direct and indirect costs from each department. Direct medical costs included those related to medications, hospitalizations, emergency room, outpatient visits, imaging and laboratory tests. Indirect costs included general and administrative expenses (eg., accounting department costs and personnel department costs), and costs incurred by non-revenue-generating areas of the hospital that must be allocated over the revenue-producing departments.

### Quality of life

The 36-item Short Form Health Survey (SF-36) is a generic quality of life questionnaire comprising 36 items/questions, distributed in eight domains, summarized in one physical and one mental component. In 2002, Brazier et al.^23^ reviewed the SF-36 and developed a new index that established a new health state classification using six domains (SF-6D). A Brazilian version of the SF-6D provides a method for generating utility values, and higher scores indicate better quality of life. The SF-6D describes health on six dimensions (physical functioning, role limitation, social functioning, pain, mental health, and vitality), from two to six seveiry levels, then describing 18,000 possible health states.^24^

### Statistical analysis

For statistical analysis of the data, quantitative variables were expressed as means ± standard deviation (SD) and qualitative variables as absolute values and percentages. Normality of quantitative data distribution was assessed using the Shapiro-Wilk test. For comparisons between groups, the unpaired t-student test was used for quantitative data, and the chi-square test for qualitative data. P-values < 0.05 were considered statistically significant. All analyzes were carried out using software SPSS Statistics, version 25.0 (IBM Corp., Armonk, NY, EUA).

## Results

### A) Sociodemographic characteristics

The study population consisted of 90 patients, 45 in each arm (warfarin vs DOACs; Table 1). The DOACs used by participants were distributed as follows: rivaroxaban 70%, apixaban 18%, dabigatran 10%, and edoxaban 2%. Risk factors and baseline conditions are described in Table 1, the mean CHA2DS2-VASc score was three and the mean HAS-BLED score was one.

**Table 1:** Sociodemographic characteristics and risk factors and baseline conditions of atrial fibrillation patients taking warfarin and direct oral anticoagulants (DOACs)

	warfarin (n = 45)	DOACs (n = 45)	p- value
Age (years)	68 ± 9	72 ± 10.5	0.07
Women (%)	51%	53%	0.08
Elementary school, n (%)	32 (71)	24 (53)	0.08 0.04
Higher education, n (%)	12 (27)	21 (47)	
Retired, n (%)	34 (76)	36 (80)	0.06
Salary, ($)	304.03 ± $211.34	379.83 ± $300.05	0.12
Need for public transportation n (%)	37 (84)	23 (52)	0.01
Travel time per visit (hr.: min.)	2:50 ± 1:24	2:55 ± 3:06	0.45
Hypertension n (%)	36 (82)	27 (60)	0.03
Diabetes mellitus n (%)	14 (31)	11 (24)	0.48
Heart failure n (%)	9 (20)	8 (17)	0.86
Myocardial infarction n (%)	6 (13)	6 (13)	0.10
Stroke/Transient ischemic attack n (%)	6 (13)	5 (11)	0.74

*Quantitative variables are expressed as mean ± standard deviation (SD) and qualitative variables as absolute values and percentages. Elementary school is defined as completion of five years of primary school. Risk factors and baseline conditions are expressed as mean and percentage. P-values < 0.05 were considered statistically significant.*

### B) Hospital, personal and total costs

Hospital costs were higher in the warfarin group (Table 2); six (12%) patients of the warfarin group and twenty-six (52%) of the DOACs routinely attended the clinic with one family caregiver. Costs related to productivity losses of patients and family caregivers are presented in Table 3 and total costs in Table 4.

**Table 2:** Hospital costs referred to the services provide for each patient

	No.	warfarin costs	No.	DOACs costs
Laboratory tests	1021	$2,347.17	671	$1,467.61
Imaging tests	208	$3,110.75	318	$5,976.94
Hospital visits	688	$12,778.02	348	$4,628.20
Emergency room	71	$9,496.54	85	$7,475.8
Hospitalizations	8	$3,355.93	10	$5,056.81
Total		$31,088.41		$24,604.74

*DOACs: direct oral anticoagulants.*

**Table 3:** Personal costs

		warfarin (45)	DOACs (45)
Patient/Family caregivers cost	Transport	$2,790.32	$1,011.10
	Food	$2,669.67	$605.25
	Drug	$1,903.27	$23,497.16
Productivity loss	Patient	$7,393.77	$5,185.43
	Family caregivers	$540.43	$628.94
Total		$ 15,297.47	$30,927.88

*DOACs: direct oral anticoagulants.*

**Table 4:** Total costs (12 months) of atrial fibrillation patients seen at the outpatient clinic of Sao Paulo Federal University in 2018 treated with warfarin (n=45) or direct oral anticoagulants (n=45)

	warfarin (45)	DOACs (45)
Personal cost	$15,297.47	$30,927.88
Hospital cost	$31,088.41	$24,604.74
Total	$46,385.88	$55,532.62

*DOACs: direct oral anticoagulants.*

### C) Overview of costs based on Brazil's unified health system (Sistema Único de Saúde [SUS]).

DOACs were 10 times more expensive than warfarin; however, the total costs of the DOACs arm were approximately 1.2 times (20%) higher. The huge price difference between these two types of anticoagulants (DOACs $23,497.16 vs warfarin $1,903.27) may be drastically reduced if we considered the other costs involved in this study. In this setting, indirect patient costs were related to loss of productivity, and direct patient costs were related to transportation and food, which contributed to increased costs observed in the warfarin arm. The difference of $9,146.74 (Formula 1) could be eliminated with a reduction of approximately 40% (Formula 2) in the price of DOACs.

**Formula 1 - Real difference: Difference between total costs with DOACs and warfarin:**

DOACs total - warfarin total

$55,532.62 - $46,385.88

Real difference of $9,146.74

**Formula 2 – Necessary reduction in DOAC price to make it comparable to the costs of warfarin:**

Price of DOACs - Real difference (formula 1) =

$23,497.16 - $9,146.74 = $14,350.42

(61% of the price of DOACs or a reduction of 39% in the drug price)

### D) Short Form SF-6D

SF-6D was used to measure the quality of life of participants (Table 5). The lowest utility scores were reported by the social functioning domain, showing an important social deficit in both arms. Mean SF-6D utility score was 0.649 for VKA and 0.641 for DOACs (Table 5). No other significant differences in health-related quality of life parameters were observed between the study arms.

**Table 5:** Mean scores of the SF-6D questionnaire domains of atrial fibrillation patients seen at the outpatient clinic of Sao Paulo Federal University in 2018 treated with warfarin (n=45) or direct oral anticoagulants (DOACs) (n=45)

	warfarin	SD	DOACs	SD
Physical functioning	0.692	0.338	0.633	0.429
Role limitations	0.564	0.356	0.570	0.364
Social functioning	0.240	0.302	0.220	0.274
Pain	0.877	0.324	0.906	0.393
Mental health	0.680	0.424	0.802	0.322
Vitality	0.446	0.236	0.448	0.220

*The SF-6D scores is calculated by perfect health rated at 1.0. SD: standard deviation; DOACs: direct oral anticoagulants.*

### Discussion

Low costs and good efficacy explain the wide use of warfarin in Brazil. However, the requirement of laboratory control and TTR variations are frequent causes of clinical complications and drug discontinuation,^25,26^ which can make patient management even more challenging.^27^ Underuse of oral anticoagulation become even more pronounced in middle-income countries, like in South American countries, where it has been reported to be less than 40%.^28^

DOACs have been introduced in Brazil since 2012^29^ with clear advantage over other therapies.^30–32^ Unfortunately, due to high costs, DOACs have been used almost exclusively used in private practice. However, the need for laboratory tests and frequent hospital visits for warfarin control create logistic problems that have become more critical in the COVID-19 pandemic due to the requirements for social distancing/isolation.^2,17,33^ These problems can be minimized with the incorporation of DOACs into Brazil's unified health system. Cost-effectiveness analysis provides evidence that, despite higher costs, DOACs are preferable and may be more cost-effective when compared to warfarin.^28,34–39^

Economic evaluation focusing only on direct financial assistance does not consider important factors for a real global analysis, like travel, food, working absenteeism, and stress.^40,41^ Warfarin group had more direct costs (e.g. food: $2,669.67 vs $605.25 for warfarin and DOACs, respectively; transport: $2,790.32 vs $1,011.10 for warfarin and DOACs, respectively) and also higher numbers of visits and laboratory tests (warfarin $1,709 vs DOACs $1,019). However, our study showed that the total costs of treatment with DOACs ($55,532.62) were higher than with warfarin ($46,385.88). This difference was directly related to the price of DOACs (DOACs $23,497.16 vs warfarin $1,903.27).

DOACs are not currently covered by the SUS in Brazil mainly because their high price. Local studies focused not only on institutional costs but also on social costs are needed. Warfarin management is difficult; general practitioners are skeptical in prescribing or maintaing AVK previous prescriptions. Our study supports that DOACs can be provided by the public health service. Silva et al.,^41^ in a study on the quality of anticoagulation control in patients with nonvalvular AF treated with warfarin in a Brazilian private care center, reported that more than 60% of the patients were below the desired target (in terms of TTR), who were associated with costs.

Based on our analyses, the “ideal” price of DOACs would be $14,350.42 (formula 2). In this situation, anticoagulation control with warfarin and DOACs would have comparable costs. Patients who do not need frequent monitoring are more prone to practice social distancing, required by health authorities during the COVID-19 pandemic.^40^ In this context, it would be highly desirable to develop a feasible approach to incorporate DOACs into the public health policy, contributing to a unified and fair health system. Reductions in the costs of DOACs group are the only possibility of cost reduction in this setting. A simple strategy for this goal would be the reduction of DOACs’ price, calculated for this purpose in this study.

Cost reimbursements in SUS are inadequate, with low federal transfer payments for public health. Thus, administrative measures are necessary to provide a rational balance of costs and affordable and conscious care. We suggested that some price interventions could generate a positive economic return and an efficient delivery of health services, and lead to a feasible scenario for the use of DOACs in clinical practice.

Interestingly, the large number of consultations did not influence the health-related quality of life of the warfarin group. Probably the high degree of humanism of our AF anticoagulation team has compensated the lack of social support and discomfort caused by repetitive hospital visits required as reported in studies from other countries.^43–45^ The provision of DOACs for patients with AF is warranted not only during a pandemic but also in normal clinical and epidemiological scenarios.

### Limitations

There were some limitations in our study. This study was not randomized and outcomes such as stroke, mortality and adherence were not analyzed. The costs were assessed during a 12-month period, and long-term follow-up may reveal more and extended benefits.

## Conclusion

The costs in the DOACs group were found to be higher than the warfarin group. Non-medical costs are an important burden in the warfarin group, requiring rational economic health strategies for disease management. A 40% reduction in the DOACs’ price may be important for the incorporation of these drugs into the SUS, a feasible policy during this COVID-19 pandemic.
